# The Effect of Oral Adenosine Triphosphate (ATP) Supplementation on Anaerobic Exercise in Healthy Resistance-Trained Individuals: A Systematic Review and Meta-Analysis

**DOI:** 10.3390/sports12030082

**Published:** 2024-03-14

**Authors:** Roberto González-Marenco, Ivonne Azeret Estrada-Sánchez, Martha Medina-Escobedo, Rodolfo Chim-Aké, Roberto Lugo

**Affiliations:** 1 School of Medicine, Autonomous University of Yucatan, Merida 97000, Mexico; rob_marenco@hotmail.com; 2 School of Sports Organization, School of Public Health and Nutrition, Autonomous University of Nuevo Leon, San Nicolas de los Garza 66455, Mexico; ln.ivonne.e@hotmail.com; 3 Research Unit, Regional High Specialty Hospital of the Yucatan, IMSS-Bienestar, Merida 97130, Mexico; marthamedinaescobedo@hotmail.com (M.M.-E.); rodolfochim@hotmail.com (R.C.-A.)

**Keywords:** adenosine triphosphate, performance-enhancing substances, muscle strength, dietary supplements, resistance training

## Abstract

Adenosine triphosphate (ATP) is an energy and signaling molecule. It is synthesized endogenously and can be taken as an oral supplement. This review aimed to identify the effects of oral ATP supplementation on anaerobic exercise in healthy resistance-trained adults. A systematic review and meta-analysis were performed based on the Preferred Reporting Items of Systematic Reviews and Meta-Analysis (PRISMA) criteria. The inclusion criteria were articles published from 2000 to 2022, with anaerobic variables (maximal strength, maximum repetitions, and maximum anaerobic power) measurable in healthy adults with experience in resistance training, only randomized placebo-controlled clinical trials (RCTs), and with the acute (a single dose 30 min to 24 h before the tests) and/or chronic (>1 day) oral supplementation of ATP. A total of five RCTs with 121 adult men were included. The oral ATP supplementation achieved significantly greater gains in maximal strength compared with the placebo (PL) (MD = 8.13 kg, 95%CI [3.36–12.90], *p* < 0.001). Still, no differences were observed in the maximum number of repetitions or the maximum anaerobic power. Furthermore, 400 mg of ATP showed improvement in anaerobic exercise regardless of the duration of the supplementation protocol. In conclusion, supplementation with 400 mg of ATP doses can improve maximal muscle strength in resistance-trained men.

## 1. Introduction

Adenosine triphosphate (ATP) is the main energy molecule in our body. It has been determined that humans have 25 mmol/kg/dry muscle of ATP, with no differences related to gender, age, or training level [1]. With creatine (phosphagen system), ATP provides enough energy to perform maximum exercises for up to 10 s, generating an insignificant energy contribution when the exercise lasts more than 30 s [2].

ATP participates during muscle contraction, reorienting the heavy heads of the thick myosin filaments and allowing cross-bridges to form with actin filaments. Subsequently, ATP is hydrolyzed through the ATPase activity of myosin, forming adenosine diphosphate (ADP) and inorganic phosphate (Pi), which causes the thin filaments to slide over the thick filaments, shortening the length of the sarcomere [3]. Likewise, ATP also participates in muscle relaxation, restoring intracellular calcium levels (Ca^2+^). The sodium/potassium pump (NA^+^/K^+^ ATPase) hydrolyzes ATP in order to move NA^+^ into the extracellular space, exchanging it for K^+^, thus causing the muscle fiber to recover its action potential and closing the Ca^2+^ channels. In addition, the sarcoplasmic reticulum has an ATP-dependent Ca^2+^ pump (Ca^2+^ ATPase), which allows for the entry of this ion into its interior [4]. The dissociation of Ca^2+^ from troponin C causes it to block the myosin binding sites, preventing the formation of cross-bridges and leading to muscle relaxation [5].

In addition to its direct intracellular involvement in muscle contraction and relaxation, it has been shown that ATP participates as a signaling molecule, with specific paracrine responses related to muscle activity [6,7,8,9]. Under hypoxic conditions, erythrocytes increase ATP synthesis, releasing it into the circulation [7]. ATP binding to purinergic receptors on the vascular endothelium appears to stimulate the production of prostaglandins-1 (PGE-1), endothelium-derived hyperpolarizing factor (EDHF), and nitric oxide, thereby resolving the relaxation of the smooth muscle fibers of the endothelium and increasing vasodilation [8,9]. Consequently, it can improve blood flow to muscle fibers, delivering more oxygen and nutrients and allowing continuous muscle action. Likewise, ATP could bind to purinergic receptors (P2Y and P2X) located in the plasma membrane, stimulating the opening of Ca^2+^ channels and its release via the sarcoplasmic reticulum [6].

As a result, oral ATP supplementation could improve physical performance during strenuous anaerobic activities, as it to known to induce hypoxia in the exercising muscle groups [10]. Studies carried out in animals have determined that, after oral supplementation with ATP, there are increases in portal vein ATP concentrations and nucleoside uptake by erythrocytes, which results in an increase in ATP synthesis by erythrocytes [11]. In this sense, greater levels of ATP and its metabolites, ADP and adenosine monophosphate (AMP), have been reported following physical activity, suggesting the major synthesis of ATP via erythrocytes in response to the hypoxic perturbations triggered by high-intensity exercise and supplementation with ATP [12]. Likewise, healthy subjects with experience in strength training can perform intense anaerobic exercises, which generate greater mechanical stress, increased temperature, and decreased intramuscular pH, which are related to a more remarkable synthesis and release of ATP via the erythrocytes, being more likely to find ergogenic effects with ATP supplementation in this population [13,14,15]. Furthermore, the tests usually implemented to evaluate anaerobic performance are biomechanically different from the movements performed in different anaerobic sports, but like the exercises performed during the sessions of a resistance-training program, allowing for a better extrapolation of the results in these subjects.

In the current literature [16,17], there is still controversy about the ability of ATP to improve physical performance, so it does not appear on the list of substances recommended by the Australian Institute of Sport. This is due, in part, to the fact that oral ATP supplementation does not result in increases in intramuscular concentrations. Furthermore, most studies do not evaluate plasma levels of ATP and its metabolites after supplementation, making it difficult to correlate changes in physical performance and body composition with supplementation [18]. Similarly, variations abound in the studies, encompassing disparate doses of oral ATP supplementation, potential coadministration with other substances, diverse levels of training or health status among subjects, protocol duration discrepancies, distinct performance variables assessed via various tests, and a lack of evaluation of changes in body composition. The presence of these variables introduces potential interference in the interpretation of the results [12,19]. For this reason, the current review and meta-analysis aimed to determine the effect of oral ATP supplementation on different anaerobic variables such as maximal strength, maximum repetitions, and maximum anaerobic power in healthy subjects trained in resistance.

## 2. Materials and Methods

### 2.1. Search Strategy

The bibliographic search was performed following the guidelines for the preparation according to Preferred Reporting Items for Systematic Reviews and Meta-Analysis (PRISMA) criteria (PRISMA checklist) [20] (Supplementary Table S1). The Cochrane Library, MEDLINE/PubMed, Dialnet, Web of Science, and ResearchGate databases were searched for articles from January 2000 to December 2022. The following keywords were taken from the Medical Subject Headings (MeSH) library and used in combination: “ATP” OR “Adenosine triphosphate” AND “Exercise” OR “Athletic performance” OR “Body composition”.

### 2.2. Selection of Studies

The titles and abstracts of all articles obtained from the initial search were reviewed individually by two authors; in the case of disagreement, a third author, who made the final decision, was consulted. The inclusion criteria of the articles were: (a) randomized clinical trials (RCTs), (b) control group with placebo (PL), (c) acute (a single dose of ATP 30 min to 24 h before the tests) and/or chronic (>1 day) oral supplementation with ATP, (d) healthy subjects trained in resistance, (e) measurement of some anaerobic performance variables, and (f) text in English and available in its entirety. The exclusion criteria were (a) research performed in vitro or in vivo, (b) the intake of ATP in combination with other substances, (c) pregnant women, and (d) letters to the editor, systematic reviews, meta-analyses, or articles which were abstracts only, with files not available or incomplete.

### 2.3. Data Extraction

The name of the first author, publication date, methodological design, age and number of participants, study duration, dose and characteristics of the ATP and the PL, performance assessment tests, and main results were extracted from each article.

### 2.4. Risk of Bias Assessment

The Cochrane risk-of-bias tool for randomized trials [21] was used. The following items were considered: random generations, allocation concealment, blinding of participants and personnel, blinding of outcome assessment, incomplete data, selective reporting, and other biases. Studies were classified as high risk of bias, low risk of bias, or unclear bias (Supplementary Table S2).

### 2.5. Study Quality Assessment

The Physiotherapy Evidence Database (PEDro) scale was used to assess the methodological quality of the studies [22]. This scale evaluates external validity (item 1), internal validity (items 2–9), and sufficient statistical information for the results to be interpretable (items 10–11). Studies with a score of 9–10 were considered to have high methodological quality; those with a score of 6–8 had good quality, while those with scores of 4–5 or 0–4 had moderate and bad methodological quality, respectively (Supplementary Table S3).

### 2.6. Statistical Analysis

Meta-analyses were performed for the different variables of anaerobic exercise evaluated (maximal strength, maximum repetitions, and maximum anaerobic power) using a random-effects model and the mean-difference (MD) method [23]. The mean values and their standard deviations (SD) were used to evaluate the effect of the variables in both groups with ATP supplementation and the PL group. The MD and 95% confidence intervals (CI) were calculated for each study and the pooled group. Values of *p* < 0.05 were considered statistically significant. Statistical heterogeneity was assessed using the *I*^2^ index. Forest plots and funnel plots (with Egger’s linear regression test) were performed using Review Manager (RevMan) statistical software version 5.4 (The Cochrane Collaboration). In addition, a meta-regression was conducted to determine the effect size changes associated with the changes in the variable responses. The regression coefficient (β coefficient) was calculated using the R program software version 4.2.2.

## 3. Results

### 3.1. Study Selection

**Figure 1:**
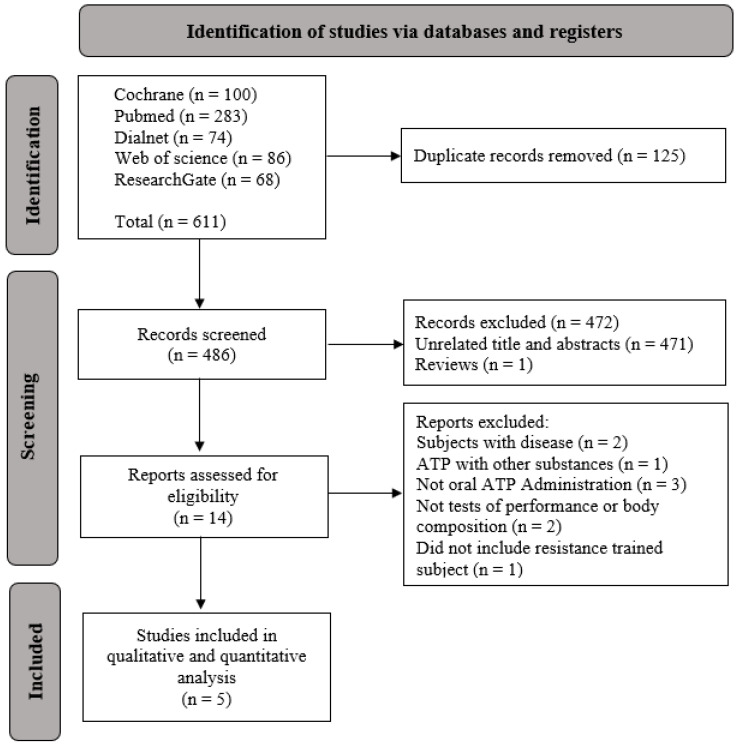
PRISMA flow diagram for the study-selection process.

The conducted literature search displayed 611 articles; 125 were eliminated as duplicate articles, 471 were unrelated to the search topic, and 1 was a systematic review. Five publications were considered for this study after applying the inclusion and exclusion criteria. Figure 1 shows the search strategy and selection of articles.

### 3.2. Study Characteristics

Studies conducted by Wilson et al. (2013) [24], Jordan et al. (2004) [17], Purpura et al. (2017) [12], Freitas et al. (2019) [25], and Dos Santos Nunes de Moura et al. (2021) [16] were selected for this study. The general characteristics of the studies are expressed in Table 1.

One hundred twenty-one men between 18 and 45 years old with experience in resistance training were evaluated. The duration of the studies ranged from 1 day (a single dose of ATP 30 min before the tests) to 12 weeks. The ingested daily dose of ATP varied from 100 to 400 mg, administered as a disodium salt in enteric-coated capsules. Capsules with rice flour, maltodextrin, or fruit-flavored powder were used as PLs. The studies evaluated maximal strength, maximum repetitions, and maximum anaerobic power as variables of anaerobic performance. The tests used to assess these variables were one-repetition maximum (1RM), with the maximum number of repetitions to exhaustion, Wingate tests, and the power of vertical jumps. In addition, only one study evaluated the changes in body composition by air dual X-ray absorptiometry (DXA) and ultrasonography [24].

All studies had a high methodological quality (Supplementary Table S3). The studies performed by Jordan et al. (2004) [17], Freitas et al. (2019) [25], and Dos Santos Nunes de Moura et al. (2021) [16] gave general instructions to maintain physical activity and diet for the duration of the studies, not maintaining strict control over these confounding variables. Conversely, in the study by Wilson et al. (2013) [24], subjects completed a 12-week periodized resistance-training protocol and followed a standardized diet designed by a sports nutritionist. Also, the studies conducted by Purpura et al. (2017) [12] and Freitas et al. (2019) [25] assessed the effect size.

### 3.3. Effect on Maximal Strength

**Figure 2:**
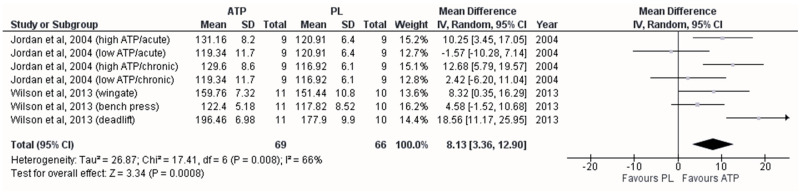
Forest plot showing differences between the effects of trials with placebo and adenosine triphosphate on maximal strength [17,24]. Numbers on the *x*-axis indicate mean differences expressed as Hedge’s g. Horizontal lines indicate the respective 95% confidence intervals.

A favorable effect on maximal strength was observed with ATP oral supplementation (MD = 8.13 kg; 95%CI [3.36–12.90]; *p* < 0.001), showing moderate heterogeneity (*I*^2^ = 66%) between the studies (Figure 2). The included pooled studies were those by Jordan et al. (2004) [17], separating their results by duration (acute = 30 min before the tests, or chronic = 14 days) and dose (low = 150 mg, or high = 225 mg) of ATP supplementation, and that of Wilson et al. (2013) [24], separating their results by the exercise test applied (bench press, deadlift, or squat). In addition, the meta-regression showed that no variable influenced the estimated effect size in this group. The funnel plot did not show publication bias (Supplementary Figure S1).

### 3.4. Effect on the Maximum Number of Repetitions

**Figure 3:**
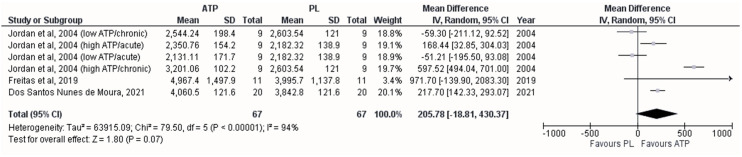
Forest plot showing differences between the effects of trials with placebo and adenosine triphosphate on maximal number of repetitions [16,17,25]. Numbers on the *x*-axis indicate mean differences expressed as Hedge’s g. Horizontal lines indicate the respective 95% confidence interval.

No effect of oral supplementation was observed between ATP or PL groups for the maximum number of repetition variables (MD = 205.78 kg; 95%CI [−18.81–430.37] *p* = 0.070), showing high heterogeneity (*I*^2^ = 94%; *p* < 0.001) between the studies (Figure 3). The pooled group was created from the studies of Jordan et al. (2004) [17], which evaluated the duration of the research and dose of ATP supplementation, Freitas et al. (2019) [25], and Dos Santos Nunes de Moura et al. (2021) [16]. Likewise, the meta-regression analysis showed that the dose of ATP (<400 mg) influenced the effect size estimate (β = −482.29; *p* = 0.028). Finally, the funnel plot showed no publication bias (Supplementary Figure S2).

### 3.5. Effect on Maximum Anaerobic Power

**Figure 4:**
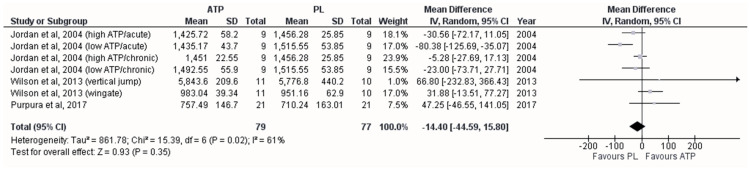
Forest plot showing differences between the effects of trials with placebo and adenosine triphosphate on maximum anaerobic power [12,17,24]. Numbers on the *x*-axis indicate mean differences expressed as Hedge’s g. Horizontal lines indicate the respective 95% confidence intervals.

The pooled group that evaluated the effect of oral supplementation in the maximal anaerobic power variable was created from the studies of Jordan et al. (2004) [17], divided into the study duration and dose of ATP supplementation, Wilson et al. (2013) [24], according to the test applied (vertical jump or Wingate test), and Purpura et al. (2017) [12]. However, Figure 4 shows that there is no effect of oral supplementation between ATP and PL groups (MD = −14.40 watts; 95%CI [−44.59–15.80]; *p* = 0.350), showing moderate heterogeneity (*I*^2^ = 61%; *p* = 0.021) between studies (*I*^2^ = 61%; *p* = 0.022). The meta-regression indicated that the duration of supplementation (acute supplementation) influenced the estimated effect size (β = −46.17; *p* = 0.047). The funnel plot showed no publication bias (Supplementary Figure S3).

### 3.6. Safety

Jordan et al. (2004) [17] reported no adverse or associated side effects after 14 days of supplementation with high or low doses of ATP; no changes in hematocrit were observed. Similarly, Wilson et al. (2013) [24] observed no significant or clinically relevant changes in blood chemistry and hematology analyses during the 12-week ATP supplementation period, with no reports of adverse effects or event. Likewise, the study conducted by Purpura et al. (2017) [12] also reported no adverse effects or events after 15 days of supplementation. For their part, Dos Santos Nunes de Moura et al. (2021) [16] evaluated the development of gastrointestinal symptoms without reporting adverse effects after acute ATP ingestion. Finally, the study conducted by Freitas et al. (2019) [25] was the only one that did not evaluate the presence of adverse effects or events in its population during the acute supplementation period.

## 4. Discussion

The Australian Institute of Sport (AIS) has developed the ABCD classification system to classify sports food and supplement ingredients into four groups based on scientific evidence and other practical considerations, determining whether a product is safe, permitted, and effective in enhancing performance sports [26]. Currently, ATP supplementation is not listed as a strategy to improve exercise performance. In fact, ATP does not appear in any of the groups that make up the list published by the AIS, even though there are clinical trials that have found ergogenic effects during exhausting physical activity following supplementation [27].

A recent review by Jäger et al. [28] identified positive effects of oral ATP supplementation on health and exercise in subjects from 7 to 65 years of age, including athletes, sedentary individuals, and people with different diseases; they concluded that ATP reduces fatigue, increases strength, improves body composition, and enhances cardiovascular health. In our study, we focused on the effects of oral ATP supplementation on anaerobic performance in healthy adult men with experience in resistance training. In addition, we performed a quantitative analysis, grouping the results according to the variable of anaerobic performance evaluated (maximal strength, maximum repetitions, and maximum anaerobic power). Moreover, we performed a meta-regression with the variables that could influence the estimated effect size.

As mentioned, the pooled analysis showed more significant gains in maximal strength when ATP was ingested than the PL (Figure 2). Interestingly, we observed that from 225 mg/day, significant improvements were reported within the ATP group (Jordan et al., 2004) [17], and with 400 mg/day, significant improvements were observed compared to the PL group (Wilson et al., 2013) [24]; hence, the dose of ATP seems decisive to achieve ergogenic effects. The gains in the maximal strength observed by Wilson et al. (2013) [24] can be attributed to the synergistic effect of combining a resistance program and chronic supplementation with ATP. During strenuous physical activities, the oxygen supply to the exercising muscle fibers decreases [29], increasing the synthesis and release of ATP via erythrocytes, generating vasodilatation through the relaxation of the vascular endothelium, and increasing nitric oxide production [30,31,32]. Also, the release of ATP via erythrocytes could participate in the repolarization of the sarcoplasmic membrane due to the action of the NA^+^/K^+^ ATPase pump and subsequent closure of Ca^2+^ channels, facilitating the coupling between relaxation and muscle contraction [33]. In this sense, it has been observed that voltage-gated CLC-1 chloride channels enhance the action of vigorous muscle action due to decreases in cytoplasmic ATP, thus decreasing muscle excitability [34]. As a result, oral ATP supplementation could improve muscle activity, either by increasing the flow of oxygen and nutrients for de novo ATP formation within muscle fibers or by crossing the sarcoplasmic membrane, exerting the effects mentioned above [35,36] and enhancing the performance in each training session.

On the other hand, these upgrades in maximal strength may be due to a potential anabolic effect of ATP supplementation. In fact, circulating increases in ATP have been observed after injury, which can directly activate the P2Y purinergic receptor, enhancing the recovery of skeletal muscle tissue [37]. Indeed, Wilson et al. (2013) [24] reported lower (*p* = 0.007) protein degradation (determined by urinary 3-methylhistidine) and greater gains in fat-free mass (4 kg vs. 2.5 kg, *p* = 0.009) and quadriceps’ muscle thickness (4.9 mm vs. 2.5 mm, *p* = 0.020) in the group supplemented with ATP compared with the PL group at 12 weeks. Furthermore, while both groups had reductions in strength during the overload phase, these were significantly lower in the group supplemented with ATP.

Also, the mechanical stress generated by resistance exercise could be related to the reported effects [38]. Studies in vitro have shown that the deformation of erythrocytes produces defects in their cytoskeleton that could positively regulate the pannexin 1 hemichannel (Panx1), which is the main ATP-release pathway [39,40,41].

In contrast, we did not find a favorable effect on maximal anaerobic power after ATP supplementation. Although Wilson et al. (2013) [24] and Purpura et al. (2017) [12] administered 400 mg/day of ATP, the difference in the results is likely due to the test type and implementation timing. Likely, a single 10 s maximal cycle ergometer set implemented by Wilson et al. (2013) [24] was insufficient to generate hypoxia, hypercapnia, or the mechanical deformation of the erythrocytes, resulting in a lack of extracellular ATP elevation [32,42]. Consequently, no effects were observed in maximum power after 12 weeks of ATP supplementation. However, favorable effects on jumping power were observed during overload weeks and at the end of the study.

On the other hand, having performed three jump tests immediately before and immediately after 10 maximum sets of 6 s on a cycle ergometer could have been too exhausting [43], overlapping the possible ergogenic effects of ATP supplementation [12]. Results in the meta-regression analysis indicated that the duration of supplementation influenced the observed effect size. In fact, the study that applied an acute supplementation protocol with the lowest doses of ATP (150 mg/day) obtained a less favorable effect for ATP [17].

In addition, we observed a positive but non-significant effect on maximum repetitions after oral ATP supplementation. Meta-regression analysis indicated that the effect size was influenced by the doses of ATP used in the studies. In this sense, it was found that doses of 225–400 mg/day achieve ergogenic effects [16,17,25]. Submaximal exercise to attenuation can increase the partial pressure of CO_2_, reduce the pH, and raise the temperature in the microcirculation of active skeletal muscle, which appear to be stimuli that upregulate the release of ATP via erythrocytes inducing the production of PGE-1, EDHF, and nitric oxide in the endothelium [8,9]. Furthermore, ATP appears to decrease sympathetic vasoconstriction by regulating α2 adrenergic receptors, potentially controlling total blood flow to the exercising muscle and local blood flow within the active muscle [44,45].

Finally, an oral intake of 150 to 400 mg of ATP proved safe in both acute and chronic protocols. In accordance with the above, Coolen et al. (2011) [46] administered oral doses of 250 to 5000 mg of ATP through enteric-coated capsules for 38 days in 32 healthy subjects, observing a significant elevation of plasma uric acid levels only with the highest dose. However, these values were within normal parameters, so they concluded that oral supplementation with ATP proved to be safe according to the hepatic and renal parameters evaluated [46].

### Strengths and Limitations

This is the first systematic review and meta-analysis to analyze the effect of ATP supplementation on anaerobic performance. Also, all included studies were double-blind RCTs with a high methodological quality. Despite the methodological variability between the included studies, we could group the results according to the evaluated anaerobic performance variable, generating more precise results. Finally, our analysis shows that the dose and duration of supplementation with ATP are variables that modify the size of the effect, observing results superior to the PL group when consuming 400 mg of ATP.

Among the limitations is that there are few articles in which the study design is randomized placebo-controlled clinical trials, maybe due to the complexity of the study and the type of participants selected; in fact, the oldest study was published in 2004. All studies included were in resistance-trained men; therefore, the results cannot be extrapolated to endurance athletes, women, or sedentary populations. In addition, only one study strictly controlled physical activity by performing a training program, and only two controlled prior food intakes using a standardized eating plan; it is necessary to maintain these variables in supplementation studies. Finally, there was moderate to high heterogeneity between the studies included.

## 5. Conclusions

Our study is the first systematic review with a meta-analysis that evaluated the effect of oral ATP supplementation on anaerobic performance in resistance-trained healthy adult subjects. The results showed that ATP supplementation induced greater gains in maximal strength than the PL group. Furthermore, ATP doses (400 mg/day) seem to be able to present an ergogenic effect in resistance-trained men. Additionally, daily intakes of 400 mg of ATP for periods of up to 12 weeks are safe in healthy subjects. The moderate to high heterogeneity observed between studies should motivate the performance of more studies with standardized methodological criteria. In the same sense, more studies that include women, sedentary populations, endurance-trained athletes, and those from different sports disciplines are required.

## Figures and Tables

**Table 1:** Characteristics of the studies included in the review.

Author/Year/Country	Study Design	Sample		Duration of the Study	Test of Performance
		ATP Doses	PL		
Jordan et al. (2004). USA [17]	RCT Double-blind	High (225 mg, n = 9) Low (150 mg, n = 9)	n = 9	21 days (14 days with supplementation)	Two tests of 30 s on a cycle ergometer at maximum intensity. 1RM bench press. Three sets of bench press (70% 1RM) to exhaustion.
Wilson et al. (2013). USA [24]	RCT Double-blind and parallel	400 mg, n = 11	n = 10	12 weeks	1RM bench press, back squat, and deadlift. Cycling test at maximum intensity for 10 s. Vertical jump Body composition by DXA and ultrasonography.
Purpura et al. (2017). USA [12]	RCT Double-blind	400 mg, n = 21	n = 21	15 days	Ten series of 6 secs on a cycle ergometer at maximum intensity. Three vertical jumps before and after the last set of cycling.
Freitas et al. (2019). Brazil [25]	RCT Double-blind, crossover	400 mg, n = 11	n = 11	One day	Four sets of half squats (80% 1RM) to failure.
Dos Santos Nunes de Moura et al. (2021). Brazil [16]	RCT Double-blind, crossover	400 mg, n = 20	n = 20	One day	Four sets of half squats (80% 1RM) to failure

## Data Availability

The datasets generated during and/or analyzed during the current study are not publicly available but are available from the corresponding author on reasonable request.

## Supplementary Materials

The following supporting information can be downloaded at: https://www.mdpi.com/article/10.3390/sports12030082/s1. Supplementary Figure S1: Funnel plot of the maximal strength; Supplementary Figure S2: Funnel plot of maximal number of repetitions; Supplementary Figure S3: Funnel plot of the maximum anaerobic power; Supplementary Table S1: PRISMA checklist; Supplementary Table S2: Summary of risk of bias: authors’ judgements about each risk of bias item for all included studies; Supplementary Table S3: Methodological quality of the studies included in the review according to the Physiotherapy Evidence Database (PEDro) scale.
