# Supplementary material for: The Effect of Oral Adenosine Triphosphate (ATP) Supplementation on Anaerobic Exercise in Healthy Resistance-Trained Individuals: A Systematic Review and Meta-Analysis
Source: Sports (Basel). 2024 Mar 14;12(3):82. doi: 10.3390/sports12030082 (PMC10975403; doi:10.3390/sports12030082)
Supplement: Supplementary file 1 [file sports-12-00082-s001.zip › Supplementary Table S2.pdf]

Supplementary Table S1. Summary of risk of bias: authors' judgements about each risk of bias item for all included studies.

Risk of bias item	Jordan et al. (2004)	Wilson et al. (2013)	Purpura et al. (2017)	Freitas et al. (2019)	Dos Santos Nunes de Moura et al. (2021)
Random sequence generation	-+	-+	-+	-+	++
Allocation concealment	-+	-+	-+	-+	++
Blinding of participants and personnel	-+	++	-+	++	++
Blinding of outcome assessment	++	++	--	--	--
Incomplete outcome data	++	++	--	++	++
Selective reporting	++	++	--	++	++
Other bias	--	--	--	--	--

(++) indicate low risk of bias; (-+) indicate unknown risk of bias; (--) indicate high risk of bias.
