# Supplementary material for: The Effect of Oral Adenosine Triphosphate (ATP) Supplementation on Anaerobic Exercise in Healthy Resistance-Trained Individuals: A Systematic Review and Meta-Analysis
Source: Sports (Basel). 2024 Mar 14;12(3):82. doi: 10.3390/sports12030082 (PMC10975403; doi:10.3390/sports12030082)
Supplement: Supplementary file 1 [file sports-12-00082-s001.zip › Supplementary Table S3.pdf]

Supplementary Table S2. Methodological quality of the studies included in the review according to the Physiotherapy Evidence Database (PEDro) scale.

Referencia	*ítems scale PEDro*											*Quality*
	1	2	3	4	5	6	7	8	9	10	11	
Jordán et al. (2004)	+	+	+	-	+	+	+	+	+	+	-	High
Wilson et al. (2013)	+	+	+	+	+	+	+	+	+	+	-	High
Purpura et al. (2017)	+	+	+	+	+	+	+	+	+	+	+	High
Freitas et al. (2019)	+	+	+	-	+	+	+	+	+	+	+	High
Dos Santos Nunes de Moura et al. (2021)	+	+	+	-	+	+	+	+	+	+	-	High

(+) indicates that it meets the criteria; (-) indicates that it does not meet the criteria.
