# Supplementary figures and images for: The Effect of Oral Adenosine Triphosphate (ATP) Supplementation on Anaerobic Exercise in Healthy Resistance-Trained Individuals: A Systematic Review and Meta-Analysis
Source: Sports (Basel). 2024 Mar 14;12(3):82. doi: 10.3390/sports12030082 (PMC10975403; doi:10.3390/sports12030082)

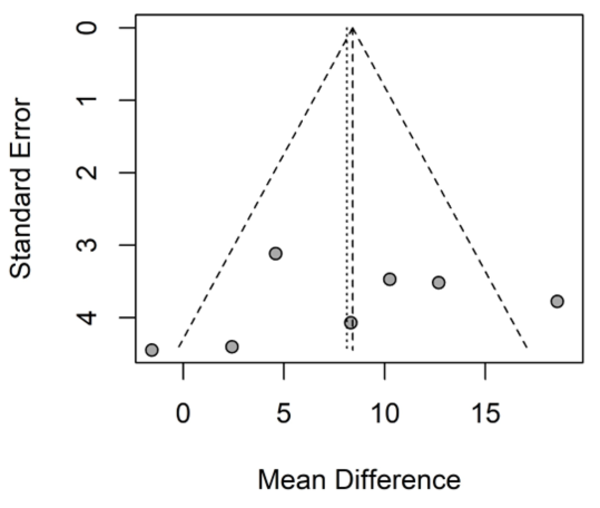

Supplement: Supplementary file 1 [file sports-12-00082-s001.zip › Supplementary Figure S1.tiff]

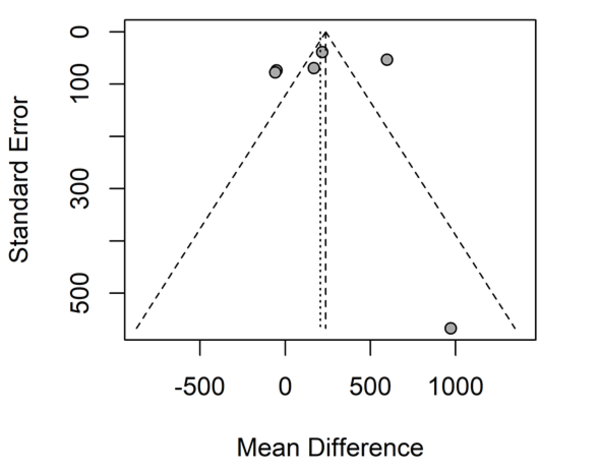

Supplement: Supplementary file 1 [file sports-12-00082-s001.zip › Supplementary Figure S2.tiff]

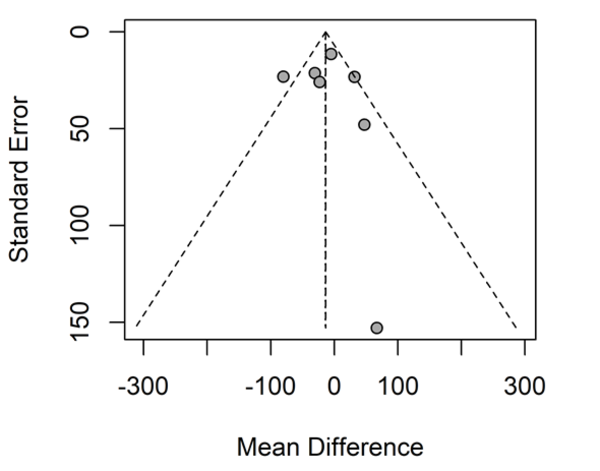

Supplement: Supplementary file 1 [file sports-12-00082-s001.zip › Supplementary Figure S3.tiff]
